# Neuroethics and AI ethics: a proposal for collaboration

**DOI:** 10.1186/s12868-024-00888-7

**Published:** 2024-08-29

**Authors:** Arleen Salles, Michele Farisco

**Affiliations:** 1 Institute of Neuroethics (IoNx), Atlanta, GA USA; 2 Centre for Research Ethics and Bioethics, Uppsala University, Uppsala, Sweden; 3 Biogem, Biology and Molecular Genetics Research Institute, Bioethics Unit, Ariano Irpino, AV Italy

**Keywords:** Neuroethics, AI ethics, Culture, Responsible conceptualization, Governance

## Abstract

The scientific relationship between neuroscience and artificial intelligence is generally acknowledged, and the role that their long history of collaboration has played in advancing both fields is often emphasized. Beyond the important scientific insights provided by their collaborative development, both neuroscience and AI raise a number of ethical issues that are generally explored by neuroethics and AI ethics. Neuroethics and AI ethics have been gaining prominence in the last few decades, and they are typically carried out by different research communities. However, considering the evolving landscape of AI-assisted neurotechnologies and the various conceptual and practical intersections between AI and neuroscience—such as the increasing application of AI in neuroscientific research, the healthcare of neurological and mental diseases, and the use of neuroscientific knowledge as inspiration for AI—some scholars are now calling for a collaborative relationship between these two domains. This article seeks to explore how a collaborative relationship between neuroethics and AI ethics can stimulate theoretical and, ideally, governance efforts. First, we offer some reasons for calling for the collaboration of the ethical reflection on neuroscientific innovations and AI. Next, we explore some dimensions that we think could be enhanced by the cross-fertilization between these two subfields of ethics. We believe that considering the pace and increasing fusion of neuroscience and AI in the development of innovations, broad and underspecified calls for responsibility that do not consider insights from different ethics subfields will only be partially successful in promoting meaningful changes in both research and applications.

## Background

The scientific relationship between neuroscience and artificial intelligence (AI) is generally acknowledged, and the role that their long history of collaboration has played in advancing both fields is often emphasized [29, 35, 91]. Beyond the important scientific insights provided by their collaborative development, both neuroscience and AI raise a number of ethical issues that are generally explored by neuroethics and the ethics of AI usually cultivated as two separate subfields of ethics.

Neuroethics can be broadly defined as a field that addresses philosophical, ethical, legal, social, and cultural questions raised by neuroscience and related technologies [27, 42, 45, 51, 60]. It emerged as a response to advances in neuroscience that have been challenging ingrained notions and related ethical views about several topics, including the structure and function of the peripheral and central nervous system, the basis of consciousness, the brain-mind relationship, and the foundations of our humanness itself.

AI ethics refers to the area of inquiry that addresses the social, regulatory, ethical, and philosophical dimensions of the development and use of AI [28]. The field attempts to formulate and develop theoretical and practical approaches to anticipate and minimize the potential adverse effects of AI research and applications across a diverse range of social and economic activities, and to enhance the advantages of AI for society.

Neuroethics and AI ethics have been gaining prominence in the last few decades, and they are typically carried out by different research communities. However, in light of evolving AI- assisted neurotechnologies and other conceptual and practical intersections between AI and neuroscience, such as the increasing application of AI in neuroscientific research, the treatment of neurological and mental diseases, and the use of neuroscientific knowledge as inspiration for AI- some scholars are now calling for a collaborative relationship between these two areas of research and practice.

This article aims to build from this proposal, seeking to further explore how a collaborative relationship between neuroethics and AI ethics can stimulate theoretical and, ideally, governance efforts. First, we begin by reviewing some reasons for calling for the collaboration of neuroethics and AI ethics. We then explore the dimensions that we think could be enhanced by the cross-fertilization between them. We believe that considering the pace and increasing fusion of neuroscience and AI in the development of innovations, broad and underspecified calls for responsibility that do not consider insights from different ethics subfields will only be partially successful in promoting meaningful changes in both research and applications.

## Main text

### Why collaboration?

While neuroethics and AI ethics have developed independently from one another, recently there have been calls for a collaborative discussion of the issues addressed by these subfields of ethics [6, 8, 26, 40, 41, 49].^1^ The need for such collaboration is grounded on the recognition of significant commonalities within the fields of neuroscience and AI: specifically, overlapping domains of research and application (i.e., shared contents), common use of fundamental concepts (i.e., shared categories), and some common fundamental concerns and challenges (i.e., shared drivers and aims).

- Overlapping domains: AI technology is increasingly assisting in several brain-related areas [93]. It is used not just to enhance neuroscientific research generating new knowledge (e.g., through optimized handling of medical data revealing new connections among them and informing more efficient predictions) but in practice for early diagnosis in brain and mental health, to improve the design and efficiency of existing neurotechnologies (e.g., Brain–Computer Interfaces (BCI)), in wearable devices for monitoring and screening brain activity, and to tailor existing drugs to the needs of individual patients, among others [69], as well as to generate new powerful bioorganic computer chips which use the computational power of the human brain, more specifically of brain organoids [13, 88].
- Common use of concepts: on one hand, AI researchers often use ontological, psychological, and normative notions and terms borrowed from neuroscience, even if occasionally these are adapted and even re-conceptualized (e.g., intelligence, consciousness, learning, neurons, synapses, among others). On the other hand, the computational metaphor about the brain (i.e., its view as an information processing device) even if less popular than in the second half of the last century [19] is still used to describe it. To illustrate, the current discussion about the possibility of conscious AI often relies on computational functionalism [12], which depicts consciousness as the result of the right computation in the right physical structure, and the brain as a particular computational system that may be replicated in AI systems.^2^
- Common or similar ethico-societal issues at the practical and theoretical levels:
  - AI and neurotechnology applications often raise questions of bias and stigma as well as concerns about their potential impact on privacy, decision making, the workplace, dual use, and human rights, among others [6, 21, 32]. Some of these risks can actually be increased by the combination of advances in neuroscience and AI.
  - AI and neurotechnology raise issues about potentially transforming the *status quo* triggering uncertainty about society and the world in the future [58].
  - Underlying many of the issues raised by the application of AI and of some neurotechnologies are philosophical convictions about the nature of humanness itself, about the line between humans and machines, and the fear of potential threats to human agency, autonomy, and dignity [8, 18, 83, 84]. This common conceptual background often results in similar drivers and aims for both neuroscience and AI as well as for the ethical reflection about them.

In view of these commonalities, bringing together the insights and developments from neuroethics and AI ethics is promising. Their collaboration can make the ethical reflection more effective (e.g., in identifying and anticipating emerging issues and in elaborating concrete and effective solutions). Indeed, below we suggest that the existing compartmentalization in addressing the issues has a negative impact on the identification and discussion of some key topics of concern. We present a few areas where collaboration promises to enrich the analysis and management of the relevant issues.

### Responsible conceptualization

While calls for responsible research and innovation have been prevalent and generally discussed in the last few years, responsible conceptualization has not been typically recognized as one of its key elements.

We characterize responsible conceptualization as the process of improving conceptual clarification in science and technology with the specific aim to enhance not just scientific research and technological development but also the ethical and normative discussion of the issues they raise as well as the determination of how to address them.^3^ The underlying idea is that clear concepts are not only a prerequisite for moving science and innovation forward: they are also key for doing so responsibly (i.e., aligning social needs and preferences with the research and innovation agenda so as to truly serve public good). Conceptual clarity enhances the identification, understanding, and management of the ethical and social issues raised by research and innovation and improves scientific communication with diverse publics.

Indeed, how concepts themselves are characterized is not only theoretically important, it is key in the communication of meanings and the narratives used to discuss science, its significance, and its outputs, and plays a big role in determining ethical priorities, citizens’ attitudes towards science and innovations, and financial support for research and innovations [4, 23, 67, 80].

Awareness of this fact is very relevant in fields like AI and neuroscience. Consider, for example, the field of AI: it uses many familiar concepts (e.g., intelligence, consciousness, autonomy, agency, learning, training, goal, reward) that because of their familiarity might seem to require no further clarification. And yet, when trying to define them, the lack of consensus regarding both what they mean, and their contextual appropriateness becomes clear. While conceptual ambiguity generally does not raise significant difficulties in day-to-day activities, vagueness may be problematic when we are trying to identify what are the societal and ethical concerns raised by AI, and how to address them productively. In fact, some of the ethical questions about harms, benefits, responsibility typically raised when discussing AI are often grounded on not fully accurate understanding of what it is and does [44].

Take, for instance, some fears and concerns raised by the possibility of conscious AI. The fact is that the conceivability of conscious AI depends on how consciousness (a controversial notion indeed) is understood in the first place [26]. If conceived as a biological phenomenon for which the biological component plays a crucial role [70, 86], then reproducing consciousness in a non-biological system would not be achievable, and ethical concerns regarding conscious AI would thus be groundless. However, if consciousness is understood within a functionalist and computational framework, its artificial implementation is conceptually consistent even if still practically not possible [20]. We would have a similar case if consciousness were conceived as a multidimensional feature, where it would be possible to artificially replicate selected dimensions [22]. These last two cases would make ethical concerns about conscious AI possibly premature and misleading, but not misplaced.

Now let’s consider an initially less controversial notion: learning. Learning is a term widely used to describe the training of artificial systems, especially Deep Learning (DL) systems (e.g., LLMs). This notion, however, becomes problematic because it may lead people to attribute other directly related features (e.g., experience, competence, flexibility, and even wisdom) to AI, features that are currently hardly applicable to AI systems. Even if there is a basic analogy between what happens in the human brain and in DL architecture when they “learn” (i.e., a change in the inter-neurons connections or weights), the two concepts do not really overlap. The fundamental difference is the level of generalization and flexibility that human learners eventually display, which goes far beyond the limited competence of DL systems, which are dependent on their training data. This fundamental difference connects to several other disanalogies, including the capacity of humans for creativity, which allows them to solve new kinds of problems they have never faced before [16].

Conceptual issues about consciousness and sentience are often also present in the field of neuroscience and in the description of some of its outputs: consider neural organoid research. The issue of consciousness, including the moral status of organoids, is a key point of discussion [38, 66, 81]. In turn, the recent controversy over the use of the term “sentience” to refer to cultures of human and mouse neurons that exhibit goal-directed activity adaptations [3, 47, 75] revolved partly around the role of conceptual clarity in interpreting research findings and the problem of ambiguous or misleading conceptualization and language in scientific progress and in the discussion of the ethical and social issues that science raises. In this specific case, the debate emerges because the term "sentience," which is inherently ambiguous [65], is often used to denote a form of consciousness that people generally regard as morally significant [52]. The need for terminological and conceptual caution in using this term is equally important in both neuroscience and AI (e.g., in the discussion about synthetic biological intelligence or artificial consciousness) [48, 85].

Technical terms can also be conceptually vague or misleading. Consider the term “digital twin” to refer to brain network models created to support diagnostic and therapeutic interventions [23, 53]. Computational brain models meet some of the basic conditions for digital twinness (i.e., a seamless connection with the physical entity that they are simulating or mirroring, a real-time data exchange between model and brain) so initially the adequacy of using the term “digital twin” might seem uncontroversial. However, ethically relevant conceptual issues still arise: computational models of the brain are not digital replicas of brains; instead they replicate very specific and targeted brain functions. As a result, their level of fidelity to their physical counterpart is more limited than the term might suggest to non-expert publics. However, if non-expert publics are aware of the creation of digital twins of the brain, they are likely to infer the existence of a type of entity that is different from what these models actually represent [23]. This is one reason for finding the use of terms such as “digital twin” to refer to these models problematic. Similar points have been made regarding the use of the term “mini brain” to refer to brain organoids [5]. Indeed, the issue of determining what level of functional and structural fidelity is required for computational models (which includes AI systems based on such models) to be considered a reliable simulation of the target object (either neural or non neural) is key and one that is shared by AI and brain research. This supports the need for collaboration when addressing this concern and the relevant ethical and social impacts.

The lack of conceptual clarity can have clear practical implications: insofar as understandings shape people’s attitudes towards scientific outcomes, the use of unclear concepts increases the likelihood of confusion, mistaken beliefs, and false expectations both within and beyond the scientific communities [4, 23].

In the last few years, the importance of attending to conceptual clarity and language in neuroscientific practices and of recognizing its role in promoting good scientific practices has been receiving some attention within the field of neuroethics. There have been concerted efforts to unveil and address conceptual issues [14, 15, 24, 27, 61, 74, 79, 87] and to identify the implications of the lack of conceptual clarity at the theoretical and practical levels. Because conceptual clarity impacts both a research lifecycle and its future applications, some neuroethicists call for using conceptual analysis to provide a semantic clarification of the relevant terms including their use and epistemic and ontological adequacy in diverse contexts [24, 27, 79]. The idea is that conceptual clarification is not just an important tool to better neuroscientific practice itself, but key to inform, refine, and enhance the ethical analysis of the issues raised by neuroscientific research and applications.

Conceptual efforts to enhance both scientific practices themselves and the discussion of the ethical issues they raise could be further advanced by collaboration between neuroethicists and AI ethicists. This could be done by, for example, doing joint work on specific concepts (e.g., intelligence, decision-making, consciousness, autonomy) that play a key role in both fields. This joint work would include reconsidering their foundation and unveiling underlying assumptions, focusing on how each field conceptualizes each notion and why, making the different conceptualizations visible, and exploring their theoretical and practical implications and ways to bridge them. This would benefit not just those who do the research (by shedding light on the epistemic justification of the concepts they use) but also non-expert publics (by enhancing their understanding of each of these disciplines and their synergies, and their capacity to assess what are the issues to address and why).

The responsible conceptualization that we have in mind requires that concepts and terminology be seen as key to scientific practice, to the interpretation of scientific findings, to the clarification of their ethical relevance and salience, and to an assessment of their desirability (e.g., anticipating their social impact). In turn, collaboration in responsible conceptualization means that conceptual clarification of some shared terms in neuroscientific and AI innovations should not be siloed and carried out in different spaces: an encompassing ethical analysis requires an overarching definition of those concepts, beyond disciplinary boundaries. Use of the relevant terms should reflect awareness of interpretations in each of the fields and awareness of how each field is using the terms, as well as awareness of possible commonalities in defining and using those terms.

### Cultural diversity

Above we proposed that responsible conceptualization will benefit from and often requires the joint conceptual work of neuroethics and AI ethics, especially for those ethically relevant concepts which are used by both fields and are therefore exposed to the risk of inconsistent understandings. Now we turn to another issue that often inadvertently shapes the identification and discussion of the ethical issues raised by innovations: cultural diversity.

For the purposes of this article, we understand culture as information (beliefs, values, assumptions) transmitted from one individual and/or group to another with an overt or covert impact on their thinking and behavior. Cultures can be demarcated by socio-anthropological factors (e.g., ethnicity, race, geographical regions) or by disciplinary or even organizational factors that often shape the understanding and perspectives of those who operate within disciplines/organizations. Here, our concern is with cultural diversity related to anthropological and sociological dimensions.

There are at least three facts that make the call for attention to culture in neuroscience and AI research and outputs compelling. First, both neuroscience and AI research and related innovations can have a significant impact on culture (including socio-political values) at the local, international, and global levels, providing new forms of knowledge, ways to interact with each other, and to think about humans and societies in general. Second, political and other non-epistemic values influence neuroscience and AI research and development, having an impact on scientific research priorities and funding, on which goals are pursued, and which technological outputs should be commercialized and could have an impact on society. Third, cultural contexts can shape how the terms used in research and the development of innovations are interpreted, as well as how the ethical and societal issues they raise are framed and addressed. In short, there is a reciprocal influence between cultural diversity on the one hand and neuroscience and AI on the other hand.

The above-mentioned three facts are ethically relevant. Regarding the first, advances in neurotechnology and AI have opened new horizons to many people, but not all. They have also raised concerns about whether the increased availability of neuro and AI innovations might increase the vulnerability of some groups, and exacerbate regional and global disparities (e.g., in terms of knowledge of and access to new technologies). The second fact appears implicitly and, sometimes, explicitly in the discussion over potential and actual bias in the design, development and deployment of AI. Within AI ethics and neuroethics the first and second aspects have been addressed in several articles and books [2, 18, 31, 39, 68–73]. Here we focus on the third which has received less attention.

What does it mean to say that cultural factors shape interpretations, framings, and conceptualization? Consider the brain: people’s understanding of what it is and does has morphed throughout history, shaped by specific historical and cultural contexts. Importantly, available technologies have played an important role in shaping the metaphors that have been used to describe and give meaning to it (e.g., in the past, the brain was explained referring to the telegraph or telephone, the most advanced technologies then available, while in more recent time we have tended to describe the brain as a computer) [17]. The same is true of the notion of mind, which underwent a long process of naturalization and whose conceptualization has been impacted by historically contingent cultural models, both from science and religion [55]. We also see cultural influence in the conceptualization of, for example, mental illness [50, 82], as well as in notions that contribute to shaping people’s view of AI, both as professionals in the field and as lay people [34].

Additionally, culture influences which scientific outputs are adopted and how they are integrated in diverse societies as well as decisions about which topics should be prioritized in ethical reflection. Indeed, lack of cultural awareness can thwart collaboration, limit the sharing of the results of neuroscientific findings and hinder awareness of the short- and long-term potential and risks of neuroscientific research (Global Neuroethics Summit [30]). And yet, some have noted that neuroethics, despite increasing attention to diversity, is rooted in Western culture and predominantly focuses on issues that affect users of new neurotechnology. This focus, however, does not adequately represent globally significant issues or alternative approaches to neurological and psychiatric health. Issues of social inequality and their impact on neurological and psychiatric health receive minimal attention and are typically not a fundamental feature of neuroethics scholarship or bioethics in general [68]. This strongly suggests that culture is a driver of ethical scrutiny by shaping what is deemed ethically problematic and determining which issues deserve ethical reflection.

The fact that interpretations, conceptualizations, and framings are informed by cultural considerations is ethically relevant not only because, as argued in the section above, they play a key role in the ethical discussion but also because they naturally influence debates on governance. Interestingly, ongoing international discussions about the governance of AI and neurotechnologies often seem to assume a culturally uniform perspective on key concepts, ethical issues, and possible solutions. There are good reasons for doing this: as the use of these innovations increases, achieving some level of alignment in ethical principles or at least agreeing on a foundational ethical framework to manage some of the questions and concerns is seen as desirable [94]. However, given the lack of cultural diversity, it is important to recognize two things. First, aspirations to universality may inadvertently mask cultural dimensions that might impact people’s understanding of the ethical issues and affect the operationalization and, ultimately, the success of proposed governance frameworks [11, 36, 78]. Second, an over emphasis on consensus of values might unintentionally lead to overlooking ethically relevant cultural contextualities and, in the worst case, might be perceived by some communities as a type of “colonialism.” Therefore, it is important to promote awareness of and sensitivity to cultural diversity and to elaborate concrete strategies for respecting such diversity. This should be done without falling into oversimplification, stereotyping, hyperbole, homologation, marginalization, idealization, trivialization, or relativism. Unsurprisingly, how to resolve the tension between the need for recognizing and attending to cultural diversity and the need for some type of global governance remains an open question, but this should not lead us to downplay either need.

Within neuroethics, a significant step toward resolving this tension includes recent proposals for a meaningful engagement with diverse publics and joint reflection on neuroethics questions (Global Neuroethics Summit [30]) across cultures. In particular, Karen Rommelfanger and Laura Specker Sullivan argue for a robust cross cultural approach that seeks to identify similarities against a background of differences, or to highlight differences against a background of similarities where the focus is put on relation [76]. In the authors’ view, this type of cross-cultural work can be carried out in different ways, from participation in multicultural meetings to research and capacity building. Importantly, we would argue that insofar as the goal is to foster intercultural understanding, identify shared concerns, enhance intra-cultural creativity, and at the same time enable a deeper understanding of the distinctive aspects of one’s culture while avoiding essentializing any one culture, attention to diverse culturally shaped conceptualizations of some of the main notions remains key.

It is true that how ethical and cultural issues might be manifested in AI ethics might be in some respects different from how they are manifested within neuroethics. To illustrate, the global landscape in neurotechnology shows that even if countries such as South Korea and China are showing an increase in development and patenting, the United States has historically led the field, ranking higher than other areas in scientific publications, investment, and patent applications [33]. Additionally, the main neurotechnology companies are located in the US and Europe (https://www.neurotech.com/charts). This would explain (although not excuse) the westernized tone of the ethical discussion. In contrast, in the context of AI research and innovation, investment is more evenly distributed across several countries in diverse regions, notably, the United States, China, United Kingdom, India, and Germany among others (https://ifamagazine.com/about/)). Accordingly, North America, Europe, and East Asia shape how the main notions are conceptualized and discussed as well as the debate over the ethics and governance of AI [64]. Still, this does not mean that all the issues are equally conceived in all places, nor that they resonate in the same way. A cross-cultural approach such as the one described is expected to facilitate a richer understanding of the cultures involved and a bridging of the dichotomous thinking often present in AI discourse frequently driven by a rhetoric that frames AI development as the next space race.

The convergence of neuroscience, neurotechnology, and AI, and the deployment of AI-assisted neurotechnologies in different parts of the world makes identification and understanding of cultural assumptions and framings particularly relevant. How these technologies are understood (e.g., their technical and social utility), which meaning people assign to them, and how they assess their societal adequacy is often not the same for every culture. In short, the place of these innovations in different societies is not solely dependent on the quality of the innovation, but shaped by local meanings, institutions, and structures. The process of identification and examination of the role that cultural contextualities play in the design and deployment of neurotechnological and AI innovations benefits from breaking down siloed approaches.

### Governance

In general, the discussion over AI governance has tended to be independent from the discussion over governance for neurotechnology. Policy-makers, academics, and several members of the AI research and user community have shown interest in addressing the governance of AI at different levels, as shown by the proliferation of both academic papers [92] and numerous AI ethics standards and recommendations [43] from international general instruments such as the OECD Recommendation of the Council on Artificial Intelligence, and the UNESCO Recommendation on the Ethics of Artificial Intelligence to codes of ethics produced by professional bodies for their members and practitioners or by other regulatory bodies such as the (IEEE Code of Ethics) as well as by recent efforts to pass laws to regulate it (The EU Artificial Intelligence Act). The existence of guidelines, recommendations, and specific attempts at regulation manifest awareness of potential and actual issues raised by AI, and some proactivity in reflecting on them and addressing how to manage them, as well as productive discussions about the challenges of implementation and of translating ethical guidelines into actionable strategies [89].

There are ethics guidance documents within the neuroscience and neurotech communities as well [6, 56, 62]. Importantly, the rapid development of diverse neurotechnologies, their wider applicability, and the recognition that the economic, social, and ethical impacts of their deployment can scale easily and outpace ethical and societal reflection [95] have led to recent calls for the development of international regulatory instruments [11, 63].

A variety of concerns have emerged in the debate over the governance of emerging technologies in general not least the issue of how to fill the gap between development and legislation [57], what kind of regulatory model to use [10, 58, 96] and which role the state should play in governance mechanisms [9]. A number of factors have been identified as important to consider in the discussion over the regulation of neurotechnology, from the issue of the philosophical, legal, and normative basis of any regulatory instrument intended to manage actual and potential risks and the need for consensus on what the goals of such regulatory instrument should be [11], to more specific concerns such as the implications of the spillover of medical neurotechnology into the consumer market, and the nature and status of neural data, its relation with mental data, and whether it requires special attention and protection.

As noted earlier, AI and neurotechnology innovations often raise many similar practical issues (e.g., autonomy, bias) and tend to generate similar philosophical concerns (e.g., identity, relation between humans and machines) even if those concerns are not identical. Let’s consider the cases of autonomy and bias. Concerning autonomy, it has been argued that it poses significant challenges in neurotechnology for a number of reasons, including that neurotechnological devices have the potential to directly access and collect neurodata and even modulate or stimulate the nervous systems, often without detection [7]. AI also raises related issues, such as relying on vast amounts of data often collected without explicit consent and possessing an impressive capacity to process that data. This capability allows AI to detect hidden patterns and access sensitive personal information that may be exploited for various purposes without the data subject’s explicit consent.

Concerning bias, its presence in the development, discovery, and interpretation of neurotechnology is widely acknowledged [32]. Neurotechnologies are often based on analysis of datasets from homogeneous populations and training samples which can skew research goals, interpretations, and assessment, while possibly leading to exclusion or misrepresentation of minority and vulnerable populations. Moreover, biased data affects what is considered "normal" brain function and the ethical implications of neuro technological advancements [7]. In turn, the prevalence of algorithmic bias within AI is also a topic of concern. A major cause of algorithmic bias is the data used to train algorithms. If historical data includes biases related to gender, race, or other factors, the algorithm can learn and continue these biases. Additionally, biases can also emerge during data collection, for example, when certain groups are underrepresented in the data, the algorithm may not perform accurately for those groups [25].

The integration of neuroscience, big data, and AI—allowing for retrospective, real-time, and predictive exploration of connections between data patterns and specific mental activities—is enhancing the development and expanding the application of various neurotechnologies (e.g., BCIs) [46]. Moreover, this convergence is intrinsically tied to the functionality of these devices. Promising as this convergence is, however, the evolving nature of both AI and neurotechnological innovations, their growing interdependence, and the continued attempt to extend their domain of application is likely to increase the emergence of some practical issues—i.e. safety, security, dual use, and privacy related issues and uncertainties- and foundational (or fundamental) issues—i.e. issues that might impact our understanding of fundamental notions (e.g., personhood, autonomy) and even our conception of human traits (such as moral thought) themselves [25]. This suggests that compartmentalized reflection on AI governance and neurotechnology governance might not be fully adequate and in fact it might be detrimental if the goal is to identify and fill regulatory gaps.

## Conclusions

While few would disagree regarding the importance of addressing the ethical issues raised by neurotechnological and AI innovations, calls for the collaboration of neuroethics and the ethics of AI are sometimes met with some puzzlement. This might be because it is sometimes difficult to determine what such collaboration means. In this paper we have attempted to shed light on such collaboration by focusing on areas that we believe would benefit from joint work: responsible conceptualization, cultural awareness, and governance discussions. We do not rule out the possibility of productive collaborative work in other areas even though more remains to be done in this respect. Importantly, we hope our reflections here will be taken as an opportunity to continue exploring the different ways in which neuroethics and AI ethics can collaborate to ethically shape our future.

## Data Availability

No datasets were generated or analysed during the current study.

## Footnotes

1 Of course, neuroethics is not the only ethics subfield that can productively collaborate with AI ethics. Computing ethics, digital ethics, information ethics, and machine ethics, among others, have bodies of work addressing ethical issues relevant to the design and deployment of AI. Importantly, however, the conceptual boundaries between Ai ethics and these other technology ethics subfields might be blurred whereas this is not the case with neuroethics which is not typically considered a type of technology ethics. For a rich discussion on the proliferation of diverse technology ethics subfields see Saetra and Danaher [77] and Llorca-Albareda and Rueda [54].

2 This view has been criticized by some researchers in neuroscience, who stress that even if the brain may be conceived as a computational system, it is organized in multiple levels and scales, which confer a high level of complexity (i.e., dynamic interaction between several components) to it [1, 90]

3 We do not engage here with an important and ultimately complementary issue that we will be addressing in a different paper: the role of conceptual engineering as a response to the conceptual disruption caused by emerging technologies (see, for example, [37, 59]). The responsible conceptualization that we propose might be seen as a component of conceptual engineering understood as conceptual adaptation (or even amelioration). In fact, responsible conceptualization proposes to adapt and/or improve our moral concepts in order to better face the specific ethical challenges arising from new technologies.
